# Long-Term Effectiveness of a Lifestyle Intervention for the Primary Prevention of Type 2 Diabetes in a Low Socio-Economic Community – An Intervention Follow-Up Study on Reunion Island

**DOI:** 10.1371/journal.pone.0146095

**Published:** 2016-01-05

**Authors:** Adrian Fianu, Léa Bourse, Nadège Naty, Nathalie Le Moullec, Benoît Lepage, Thierry Lang, François Favier

**Affiliations:** 1 Centre d’Investigation Clinique de la Réunion (CIC 1410), Institut National de la Santé et de la Recherche Médicale (Inserm), Saint-Pierre, la Réunion, France; 2 Centre d’Investigation Clinique de la Réunion (CIC 1410), Centre Hospitalier Universitaire la Réunion (CHU la Réunion), Saint-Pierre, la Réunion, France; 3 Unité Mixte de Recherche 1027 (UMR1027), Université Toulouse III Paul-Sabatier, Toulouse, France; 4 Unité Mixte de Recherche 1027 (UMR1027), Institut National de la Santé et de la Recherche Médicale (Inserm), Toulouse, France; 5 Service d’Endocrinologie, Diabétologie et de Nutrition, Centre Hospitalier Universitaire la Réunion (CHU la Réunion), Saint-Pierre, la Réunion, France; 6 Centre Hospitalo-Universitaire Toulouse, Toulouse, France; Medical University Innsbruck, AUSTRIA

## Abstract

In type 2 diabetes (T2D) prevention research, evidence for maintenance of risk factor reduction after three years of follow-up is needed. The objective of this study was to evaluate the long-term effectiveness of a combined lifestyle intervention aiming at controlling body weight (BW) and waist circumference (WC) in non-diabetic, overweight/obese adults living in a low socio-economic community. On Reunion Island, 445 adults living in deprived areas, aged 18–40 and at high-risk for T2D, were included in an intervention versus control trial for primary prevention (2001–2002). The intervention promoted a healthy diet and moderate regular physical activity, through actions strengthening individuals or community and improving living conditions. The control group received a one-shot medical information and nutritional advices. After the end of the trial (2003), 259 of the subjects participated in a follow-up study (2010–2011). The outcomes were the nine-year changes from baseline in BW, body mass index (BMI) and WC measurements, separately. Statistical analyses were performed on an intention-to-treat basis, using available and imputed datasets. At inclusion, T2D risk factors were prevalent: family history of diabetes in first-degree relatives (42%), women with a personal history of gestational diabetes (11%), total obesity (43%, median BMI 29.1 kg/m²) and central obesity (71%). At follow-up, the adjusted effect on imputed dataset was significant for WC -2.4 cm (95% confidence interval: -4.7 to -0.0 cm, p = 0.046), non-significant for BW -2.2 kg (-4.6 to +0.2 kg, p = 0.073) and BMI -0.81 kg/m² (-1.69 to +0.08 kg/m², p = 0.074). A specific long-term effect was the increased likelihood of reduction in adiposity: BW loss, BMI reduction, and WC reduction were more frequent in the intervention group. In the context of low socio-economic communities, our data support the assumption of long-term effect of lifestyle interventions targeting total obesity and central obesity two major drivers of T2D.

## Introduction

In the past several decades, diabetes has reached epidemic proportions worldwide [1], and most specifically type 2 diabetes (T2D) which accounts for about 90–95% of diabetes cases [2]. This widespread phenomenon has been shown to be related to fundamental societal determinants of health at population level mediated by physical inactivity, unbalanced diet and obesity [3].

These modifiable risk factors have been targeted by randomized prevention trials in the USA, China, India, Japan and Finland [4], which demonstrated that lifestyle interventions can reduce incidence or delay development of T2D. Health benefits could remain for up to fourteen years after the end of the intervention, compared with the control situation [5–8].

As many of these clinical trials were conducted in resource-intensive settings [9] with selected volunteers, translation studies have been implemented in real-life settings to evaluate the effectiveness of such programs. The most recent meta-analysis in this pragmatic framework showed that exposure to lifestyle intervention results in a 2.32 kg mean weight loss after 12 months (95% confidence interval: -2.92 to -1.72 kg) [10]. The authors concluded that more research was needed to describe long-term maintenance of weight loss and diabetes-prevention effects.

To date, research in T2D prevention interventions is still needed [11]. From the literature, four recommendations were found to emerge: I- start lifestyle intervention early with a primary prevention approach [12]; II- implement community-based efforts in real-life settings [11]; III- focus on vulnerable groups such as communities with low socio-economic status [13,14]; IV- study duration of effect [10,14]. Indeed, very few studies have evaluated long-term benefits of T2D prevention after lifestyle intervention discontinuation, beyond three years of follow-up [5,7,8,15]. To the best of our knowledge, none of the published studies addresses these four research points together.

Following these recommendations, our objective was to evaluate the long-term effectiveness of a combined lifestyle intervention for controlling body weight (BW) and waist circumference (WC) in non-diabetic, overweight or obese adults living in a low socio-economic community.

## Methods

### Study design and settings

We used the REDIA-prev1 cohort study (acronym for REunion DIAbetes primary prevention), an *intervention follow-up study* [16] (see detailed description below) setting on Reunion Island over 2001–2011.

Reunion Island is a French overseas territory of ~800,000 inhabitants; it is located in the South-West Indian-Ocean, and displays a huge T2D prevalence rate in the general population aged 30–69 (20.1%, 95%CI: 18.7–21.4). A description of this health situation, illustrating epidemiological transitions and westernization of lifestyles over the past few decades, has already been published by our team [17].

The first component of the REDIA-prev1 cohort study was the lifestyle intervention controlled trial (i.e., a quasi-experimental design) which has been described elsewhere [18]. It was implemented in 2001–2003 to evaluate the short-term effectiveness of a combined lifestyle intervention for one-year weight reduction in T2D high-risk adults living in a low socio-economic area. For this purpose, two districts (Basse Terre–Joli Fond (~6,200 inhabitants), and Ravine des Cabris (~11,400 inhabitants)) within the municipality of Saint-Pierre (~69,000 inhabitants) were identified as vulnerable according to census statistics [19,20], and were chosen for socio-demographic comparability and geographical convenience. One of the districts was used for selection of the intervention group, while the other was used for selection of the control group. These groups were formed by restriction of eligibility [16] to *high-risk* subjects (see definition below) screened and enrolled at home.

The second component of the design was the follow-up, implemented in 2010–2011, which assessed long-term changes in BW, body mass index (BMI) and WC nine years after inclusion.

### Population

The target population eligible to the lifestyle intervention trial was composed of men and women (non-pregnant), aged 18–40, with no reported serious illness (e.g., diabetes, cardiovascular disease, cancer) nor disability (incompatible with physical activity), living in the studied districts, and screened at home by medical staff as *high-risk* subjects. This status was based on a combination of risk factors [18]: total obesity (BMI ≥ 30 kg/m²) or overweight status (25 ≤ BMI < 30 kg/m²) associated with at least one secondary risk factor, or central obesity [21] (WC ≥ 100 cm for men, ≥ 90 cm for women). The secondary risk factors were: treated or screened high blood pressure (≥ 140/90 mmHg), elevated glycated haemoglobin A1c (HbA1c = 5.5%-5.9% [22]), a family history of diabetes in first-degree relatives, and for women with a personal history of gestational diabetes and/or having a child whose birth weight was greater than or equal to four kg. On Reunion Island, central obesity was found to be one of the morphological characteristics most closely linked with T2D [17].

All subjects were screened as non-diabetic based on HbA1c measurements (immunological method, DCA 2000®) < 6.0% [22] (cut-off inferior to medical guidelines in 2010: 6.5% [2]).

All subjects included were eligible for the follow-up except those living outside Reunion Island or having a serious disability that created real difficulties in data collection.

### Intervention

The weight-reduction program implemented in the intervention group lasted approximately one year. Its goal was to experiment with methods for promoting individual changes in nutrition behaviour (i.e., healthy diet and moderate regular physical activity). The description of the intervention implementation was published previously [18]. In the district in which the intervention took place, high-risk persons were informed of the workshops during screenings, with reminders sent out by mail or given via telephone. A room was made available by the City of Saint-Pierre; in it, ergonomic exercise bicycles, rowing machines, and treadmills, as well as a fully equipped kitchen, were set up. The intervention adopted a community health approach using peer education. A team consisting of a sports coach, a prevention coordinator trained in dietary/nutritional health and support group management, and three assistant coordinators, all creole and residing in the neighborhood (within intervention district), to run the workshops. The participants were invited to propose activities (for example, recreational activities). To facilitate enrolment in the program, we gave family members and friends the option of accompanying the participants, and created a playspace and games library for children, so that parents can do indoor physical activity or go out walking. Neighborhood organizations were informed of our activities, and certain of them were stakeholders in the program, depending on their particular areas of focus (walks and hikes, arranging walking routes in the neighborhood). The neighborhood fruit and vegetable merchant agreed to offer substantial discounts to anyone presenting an intervention participant’s card.

The workshops were organized around three main themes: *i-* how to eat a balanced diet: nutritional information followed by practical learning via breakfast and cooking workshops coupled with communal meals—all based on eating as many vegetables as one likes, plus fruit, dairy, and fish, and reducing caloric intake (limiting oil, trimming the fat from meat, reducing the amount of rice eaten); *ii-* indoor physical activity using ergonomic/easy-to-use machines (the sports room was open every day from 8 AM to 7 PM, and Saturdays from 9 AM to 12 PM, with the only limit being the number of participants) that recorded energy expenditure and length of effort; group walks around the neighborhood (two hours long on average, three times a week, 68 sessions in total) and hikes (nine of them, four to six hours long, occurring roughly once a month); recreational activities one or twice a week (21 dance and 49 basketball sessions in total); *iii-* support groups that allow participants to express their questions on nutrition, physical activity, the body and health (six in total). Participation in these different workshops was unrestricted and free of charge.

Thus, according to the classification of Mrs. Margaret Whitehead [23], this complex intervention [24] involved three categories of health action: I- strengthening individuals (screening at home for the T2D risk factors, immediate delivery of medical information and guidelines to high-risk subjects, increasing self-esteem with support groups, learning by practice in workshops); II- strengthening community (peer education, implication of participants in the intervention process, use of associative local network, targeting a group dynamic, dealing with local linguistic context and difficulties of expression); III- improving living conditions (provision of an open intervention room with accessible hours and childcare, facilitation of urban walking, improvement of local food supply). In the logic of this intervention, half of the combined theoretical components dealt with strengthening individuals.

In the control group, high-risk subjects received medical information and nutritional advice (written/oral) just after the screening.

### Data collection

The follow-up data were collected at home in two visits: first, by one research-skilled nurse with an assistant, doing medical examination to assess T2D risk factors, lifestyle and socio-demographic characteristics; second, by a dietician collecting data on physical activity and diet (usual physical activity and usual diet, history of physical activity and diet since trial completion), using face-to-face questionnaires.

#### Anthropometric measurement

The same process of anthropometric measurement was applied in the two groups, at inclusion and at follow-up: that is, assessment by a mobile medical staff, at home, in the morning, of subjects lightly clad, shoeless, with empty bladder. Height was measured at inclusion in standing position using a rod. BW was measured using a portable scale Seca® (minimum graduation = 1.0 kg) at inclusion; Tanita® (minimum graduation = 0.1 kg) at follow-up. At inclusion and follow-up, WC was measured two times, with a tape, to the nearest centimetre, in the standing position midway between the tip of the iliac crest and the lowermost rib, during minimal respiration. Pregnancy and breastfeeding were two non-eligible conditions for BW and WC measurements. BMI was calculated as BW (in kilograms) divided by height squared (in meters), and categorized according to WHO cut-off points [25].

#### Usual diet assessment

For usual diet assessment, the interview covered food consumption over seven days, with food portion size estimated by a photo album. Data were recorded in GENI v6.5 software (produced by Micro6) to calculate the average daily intake (total energy, nutrients) using the REGAL food composition table. Individual data (physical activity level, sex, age, body weight and height [26]) were used with the Goldberg cut-off method [27] to assess the quality of reporting. Statistical analysis on a subset of 175 subjects with available data showed that 29% of subjects underreported total energy intake and 2% overreported. This level of misreporting is concordant with previous review: 30% prevalence on average [28]. In our study, the likelihood of this information bias seemed to be higher in the control group than in the intervention group (reference category): the prevalence ratio of underreporting (adjusted for gender, age, educational level, BMI, smoking, number of meals per day, and reported nibbling) was 1.60 (95% CI: 0.93 to 2.46). Because of this differential misclassification, we have thus decided not to publish results from the evaluation of nutrient intake reported at follow-up.

### Outcomes

The continuous outcomes were the nine-year changes (i.e., follow-up measurement minus inclusion measurement) in BW (kg), BMI (kg/m²) and WC (cm), separately. The individual mean of the two successive WC measurements was used to calculate the nine-year change. The continuous outcomes permitted the generation of the binary outcomes (BW loss, BW loss ≥ 5%, BMI reduction and WC reduction). BW loss ≥ 5% used inclusion BW as a reference.

### Statistical analyses

**Fig 1:**
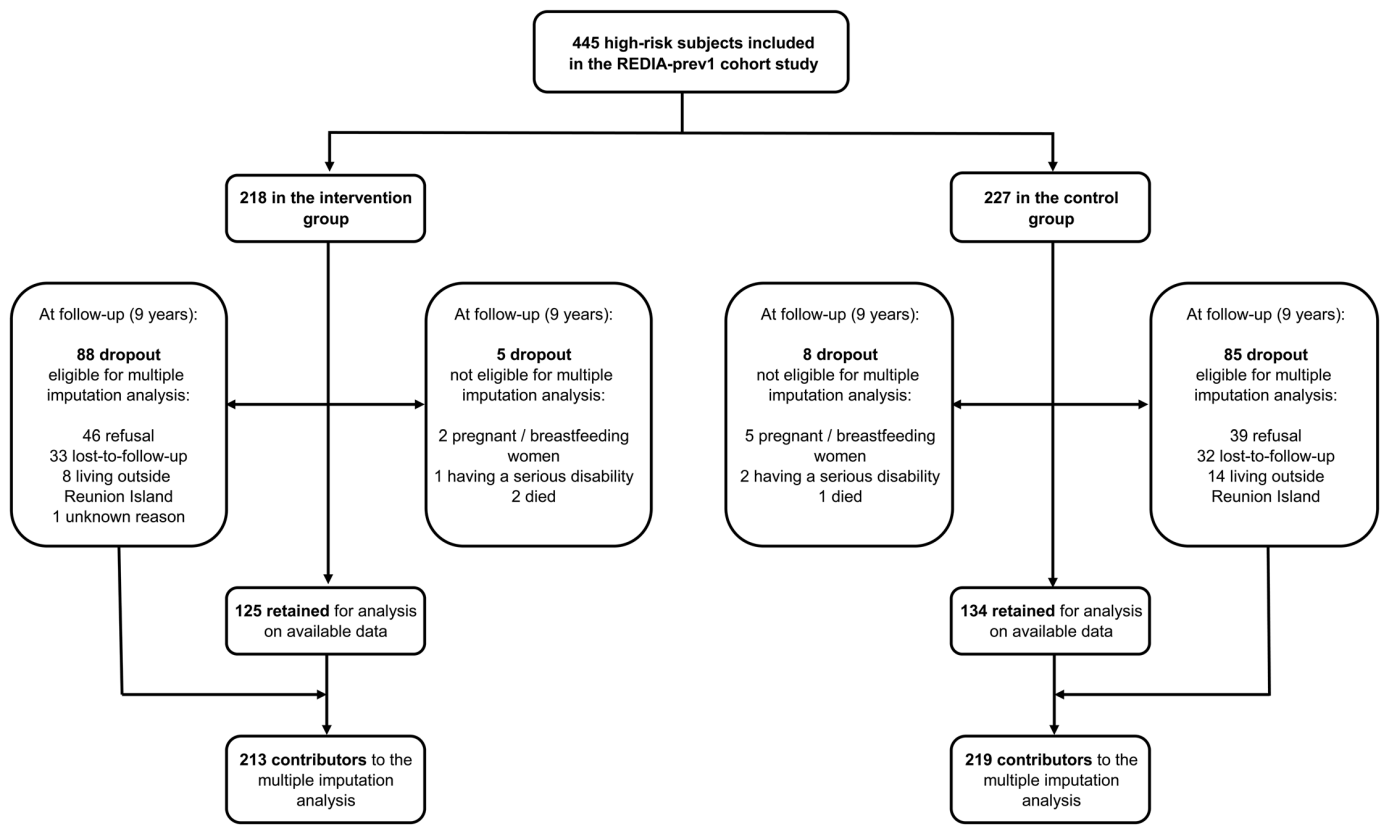
Selection of the participants for the long-term evaluation analyses in the REDIA-prev1 cohort study. (Reunion Island, 2001–2011).

Descriptive statistics included number, proportion (Pr), percentage, mean, standard error, median, interquartile range, and minimum, maximum. Comparisons between independent samples used chi-square tests for categorical variables, and Student’s t-tests or Wilcoxon rank-sum tests, as appropriate, for quantitative variables. The intervention’s long-term effect was evaluated with an intention-to-treat analysis. Two methods were applied: I- for continuous outcome, the intergroup difference in mean change (Δ = intervention minus control); II- for binary outcome, the relative risk (RR) associated with a decrease in change (intervention as exposure category versus control as reference category). In multivariate analysis, estimates were adjusted on baseline characteristics (i.e., characteristics collected at inclusion): outcome measurements, HbA1c, characteristics the distribution of which significantly differed between the two groups, and characteristics linked to attrition, in order to make the missing at random assumption (MAR) more plausible [29]. Multivariate analyses were conducted with analysis of variance (ANOVA) models for continuous outcomes, and Poisson modified regression models for binary outcome [30]. Analyses on the available dataset were carried out with SAS version 9.2 (SAS Institute Inc., Cary, NC, USA). To minimize selection bias occurring due to attrition of the cohort, missing data were managed under MAR with multiple imputation by chained equation (MICE, ice and MIcombine packages, Stata version 10, StataCorp, College Station, Texas, USA). Three categories of dropout were excluded prior to the multiple imputation process (Fig 1), because of the incident-modified nutritional status at follow-up (expected to be independent of exposure to the intervention): pregnant or breastfeeding women, people with a serious disability, and deaths, all three categories accounting for a total less than 3% of the entire cohort. Variables included in the imputations models were: the baseline characteristics, the exposure-group, participation in the medical visit at trial completion (yes/no), and the shifted log transformation of BW and WC at follow-up, respectively [31]. Multiple imputation programs make no distinction between independent and dependent variables of the multivariate model of interest [32]. Forty imputed datasets [31] were generated from an original dataset of 432 observations. Algorithm convergence was checked by graphical output. Before pooled analysis, BW and WC were back-transformed to their original scale [31]. Statistical significance level was set to 5%.

### Minimum detectable difference: prior calculation

With 100 high-risk subjects per group it would be possible to detect a minimum intergroup absolute difference in BW mean change of 2.0 kg, assuming standard deviation 5.0 kg, statistical power 80% and two-sided alpha 5%.

### Ethical considerations

The REDIA-prev1 cohort study followed French law and received approval from the regional ethics committee “CPP Sud-Ouest et Outre Mer III” (No. 2010/56). All subjects gave their free, written, informed consent to participate in the research study.

## Results

### Selection of participants

Among the 445 participants enrolled in the cohort (Fig 1), 259 (58%) were followed up on between October 2010 and April 2011. This rate was comparable between intervention group and control group, respectively 57% and 59% (p<0.72). The follow-up length (median 9.0 years) was three months higher (p<0.001) in the intervention group (median 9.1 years, min-max 8.1–9.6) than in the control group (median 8.9 years, min-max 8.5–9.3). Within the intervention group (Table 1), compared to dropouts, followed-up participants were older (respectively, median 31.7 years, interquartile range [27.4–35.1] vs. 33.4 years [29.2–37.3], p<0.04) and more often women (63% vs. 77%, p<0.04). In both exposure groups, the mean baseline level of BW, BMI and WC did not significantly differ between followed-up and dropout samples, nor for family history of diabetes, HbA1c, high blood pressure or lifestyle characteristics (Table 1 and Table 2).

**Table 1:** Baseline characteristics of the high-risk subjects according to exposure-group and follow-up status.

Socio-demographic and lifestyle characteristics at inclusion	Intervention group		Control group	
	Followed up	Dropout	Followed up	Dropout
N	125	93	134	93
Age (years)	33.4 ^a^	31.7 ^a^	32.3	31.0
	[29.2–37.3]	[27.4–35.1]	[26.9–37.2]	[25.5–36.5]
Gender				
Women	96(77) ^a^	59(63) ^a^	95(71)	67(72)
Men	29(23)	34(37)	39(29)	26(28)
Occupation								
Yes	51(41)	31(33)	38(28)	26(28)
No	74(59)	62(67)	96(72)	67(72)
Smoking								
Past or never	100(80)	72(77)	100(75)	65(70)
Current	25(20)	21(23)	34	(25)	28(30)
Occupational physical activity								
Important or very important	54(43)	46(49)	53(40)	30(32)
Low or moderate	71(57)	47(51)	81(60)	63(68)
Stress feeling								
Never or seldom	74(59)	52(57)	96(72)	61(66)
Often or very often	51(41)	40(43)	38(28)	32(34)

^a^ indicates statistical significance (p<0.05) when testing difference between Followed up sample and Dropout sample within group.

Data are: n (%), median [interquartile range], mean ± standard error.

**Table 2:** Baseline characteristics of the high-risk subjects according to exposure-group and follow-up status *(Continued)*.

Risk factors at inclusion	Intervention group		Control group	
	Followed up	Dropout	Followed up	Dropout
N	125	93	134	93
BW (kg)	77.9 ± 1.3	79.4 ± 1.5	79.8 ± 1.2	79.5 ± 1.5
WC (cm)	97.4 ± 0.9	98.0 ± 1.0	96.7 ± 0.9	96.4 ± 1.0
BMI (kg/m²)	29.3	29.0	29.1	29.4
	[26.8–32.5]	[26.8–32.4]	[27.0–32.4]	[27.1–32.0]
BMI class				
18.5–24.9 kg/m²	5(4)	3(3)	2(2)	2(2)
25–29.9 kg/m²	65(52)	54(58)	74(56)	48(52)
≥ 30 kg/m² (Total obesity)	55(44)	36(39)	56(42)	43(46)
Central obesity ^b^								
No	35(28)	34(37)	39(29)	28(30)
Yes	90(72)	59(63)	95(71)	65(70)
High blood pressure ^c^								
No	79(64)	52(56)	70(53)	52(57)
Yes	45(36)	41(44)	63(47)	40(43)
HbA1c								
< 5.5%	96(77)	72(77)	94(70)	55(59)
5.5–5.9%	29(23)	21(23)	40(30)	38(41)
Family history of diabetes in first-degree relatives ^d^								
No	75(60)	48(52)	76(57)	51(55)
Yes	50(40)	45(48)	58(43)	42(45)
For women having children:								
Personal history of gestational diabetes								
No	64(84)	51(94)	80(94)	57(90)
Yes	12(16)	3(6)	5(6)	6(10)
Having a child with birth- weight ≥ 4 kg								
No	66(87)	52(96)	77(91)	58(92)
Yes	10(13)	2(4)	8(9)	5(8)

^b^ based on WC using two gender specific cut-off, 90 cm for women, 100 cm for men.

^c^ treated or screened if not treated (blood pressures ≥ 140/90 mm Hg).

^d^ father / mother / brother / sister / child.

BMI = body mass index. BW = body weight. HbA1c = glycated haemoglobin A1c. WC = waist circumference.

This high-risk population (n = 259) comprised adults aged 18–40 years, mostly women (74%), with prevalent risk factors: family history of diabetes in first-degree relatives (42%), total obesity (43%, median BMI 29.1 kg/m²), central obesity (71%) and high blood pressure (42%). Within the sub-population of women having children (n = 161), 11% reported a personal history of gestational diabetes (same figure for those having a child with birth-weight ≥ 4 kg).

When comparing the followed-up subjects (Table 1), participants of the intervention group reported having an occupation as well as feelings of stress more often than those of the control group (both 41% versus both 28%, p<0.04 for all comparisons).

### Rationale for the MAR assumption in cohort attrition at follow-up

To assess the short-term effectiveness of the lifestyle intervention, the design of the REDIA-prev1 cohort study involved a medical examination at trial completion. In Table 3, the subjects who had participated in the trial completion examination were given a follow-up exam (seven years later on average) with a rate more than twice that of the non-participants: respectively, 66% versus 32% (p<0.001).

**Table 3:** Participation in trial completion examination predictive of follow-up participation.

	Participation in follow-up		
Participation in trial completion examination	Yes	No	p
**Yes**	236 (66)	123 (34)	<0.001
**No**	23 (32)	50 (68)	

Data are: number (row percentage). Pvalue was calculated on the sample of subjects contributors to the multiple imputation analysis (N = 432; see Fig 1), using Chi-square test (1 df).

### Nine-year changes in BW, BMI and WC: continuous outcomes

On average, BW, BMI and WC increased between inclusion and follow-up, in both exposure groups (Table 4).

**Table 4:** Nine-year changes in body weight (BW), body mass index (BMI) and waist circumference (WC): adjusted estimates (mean, Δ, RR).

Outcomes	Analysis on dataset	Intervention group				Control group				Intention-to-treat analysis: intervention versus control			
*Continuous*		N	Mean	95% CI	p	N	Mean	95% CI	p	N	Δ	95% CI	p
**BW (kg)**	available	125	+3.1	+1.3 to +4.8	0.001	134	+5.1	+3.5 to +6.8	<0.001	259	-2.1	-4.1 to -0.1	0.043
**BW (kg)**	imputed	-	+3.1	+1.5 to +4.7	<0.001	-	+5.3	+3.6 to +7.0	<0.001	-	-2.2	-4.6 to +0.2	0.073
**BMI (kg/m²)**	available	125	+1.11	+0.44 to +1.77	0.002	132	+1.90	+1.26 to +2.54	<0.001	257	-0.79	-1.57 to -0.01	0.046
**BMI (kg/m²)**	imputed	-	+1.19	+0.59 to +1.79	<0.001	-	+2.00	+1.37 to +2.63	<0.001	-	-0.81	-1.69 to +0.08	0.074
**WC (cm)**	available	124	+1.9	+0.1 to +3.7	0.043	134	+4.8	+3.0 to +6.5	<0.001	258	-2.9	-5.0 to -0.8	0.008
**WC (cm)**	imputed	-	+2.1	+0.3 to +3.9	0.022	-	+4.5	+2.8 to +6.1	<0.001	-	-2.4	-4.7 to -0.0	0.046
*Binary*	**Dataset**	**n/N**	**Pr**	**95% CI**	**p**	**n/N**	**Pr**	**95% CI**	**p**	**N**	**RR**	**95% CI**	**p**
**BW loss**	available	40/125	0.32	0.24 to 0.41	<0.001	28/134	0.21	0.14 to 0.29	<0.001	259	1.60	1.04 to 2.46	0.032
**BW loss**	imputed	-	0.35	0.27 to 0.43	<0.001	-	0.25	0.17 to 0.32	<0.001	-	1.50	1.02 to 2.22	0.040
**BW loss ≥ 5%**	available	21/125	0.17	0.11 to 0.25	<0.001	12/134	0.09	0.05 to 0.15	<0.001	259	1.83	0.90 to 3.71	0.096
**BW loss ≥ 5%**	imputed	-	0.19	0.13 to 0.26	<0.001	-	0.12	0.06 to 0.18	<0.001	-	1.66	0.91 to 3.02	0.100
**BMI reduction**	available	40/125	0.32	0.24 to 0.41	<0.001	27/132	0.20	0.14 to 0.28	<0.001	257	1.61	1.04 to 2.50	0.032
**BMI reduction**	imputed	-	0.35	0.27 to 0.43	<0.001	-	0.24	0.17 to 0.32	<0.001	-	1.49	1.01 to 2.20	0.046
**WC reduction**	available	55/124	0.44	0.35 to 0.54	<0.001	36/134	0.27	0.20 to 0.35	<0.001	258	1.60	1.13 to 2.25	0.007
**WC reduction**	imputed	-	0.44	0.36 to 0.52	<0.001	-	0.30	0.22 to 0.37	<0.001	-	1.46	1.06 to 2.00	0.019

95% CI = 95% confidence interval. Pr = univariate proportion (= n/N). RR = adjusted relative risk (intervention group as exposure category versus control group as reference category). BW loss ≥ 5% of the inclusion measurement. Continuous outcome within group = follow-up measurement minus inclusion measurement. Δ = intergroup difference in mean change (intervention minus control). p for test with (H_0_: mean = 0) or (H_0_: Δ = 0) or (H_0_: Pr = 0) or (H_0_: RR = 1) according to outcome and comparison. All results, except Pr, were adjusted on baseline characteristics (gender, age, BW or BMI or WC, HbA1c, occupation, stress). Missing data were imputed under MAR assumption with MICE method implemented in Stata 10 (ice and Micombine packages). Variables included in the imputation models: baseline characteristics, exposure-group, participation in the medical visit at trial completion (yes/no), and the shifted log transformation of BW and WC at follow-up, respectively. Forty imputed datasets were generated from an original dataset of 432 observations.

Based on available dataset, adjusted mean change in BW was +3.1 kg (p = 0.001) in the intervention group versus +5.1 kg (p<0.001) in the control group; adjusted mean change in BMI, was +1.11 kg/m² (p = 0.002) in the intervention group versus +1.90 kg/m² (p<0.001) in the control group; adjusted mean change in WC was +1.9 cm (p = 0.043) in the intervention group versus +4.8 cm (p<0.001) in the control group.

Based on imputed dataset, adjusted mean change in BW was +3.1 kg (p<0.001) in the intervention group versus +5.3 kg (p<0.001) in the control group; adjusted mean change in BMI was +1.19 kg/m² (p<0.001) in the intervention group versus +2.00 kg/m² (p<0.001) in the control group; adjusted mean change in WC was +2.1 cm (p = 0.022) in the intervention group versus +4.5 cm (p<0.001) in the control group.

Looking at the long-term effect, the statistically significant inter-groups difference (intervention minus control) was for WC: -2.9 cm (p = 0.008) using available dataset, and -2.4 cm (p = 0.046) using imputed dataset.

In Table 4, all estimates (except Pr) were adjusted on baseline characteristics: gender, age, occupation, stress, HbA1c, BW or BMI or WC. Non-adjusted analyses are presented in S1 Table.

### Nine-year changes in BW, BMI and WC: binary outcomes

Within the intervention group (Table 4), depending on the dataset used for the statistical analysis, the proportion of subjects whose BW decreased between inclusion and follow-up was in the range of 0.32–0.35 (same figures for BMI reduction). The proportion of subjects who had a WC reduction was 0.44 whatever the dataset used.

Within the control group (Table 4), depending on the dataset used for the statistical analysis, the proportion of subjects whose BW decreased between inclusion and follow-up was in the range of 0.21–0.25 (0.20–0.24 for BMI reduction), and the proportion of subjects who had a WC reduction was in the range of 0.27–0.30.

Proportions in both groups were different from 0 (p<0.001 for all proportions).

Looking at the long-term effects (Table 4), BW loss, BMI reduction, and WC reduction were more frequent in the intervention group than in the control group (adjusted RR > 1.00 statistically significant), using either available dataset or imputed dataset.

Non-adjusted RR are presented in S1 Table.

## Discussion

Our cohort study, implemented in a low socio-economic community, is innovative research in the field of primary prevention of T2D, with post-intervention data used to estimate long-term effects of prevention. The findings support that initiation of individual lifestyle modifications over a short time (one year) could continue to have a preventive impact for up to nine years after inclusion on WC increase, in non-diabetic, overweight or obese adults with central obesity. Elevated WC is an important predictive factor of T2D [33]. Furthermore, the intervention may provide specific long-term health benefits for a part of the targeted population. Indeed, BW loss, BMI reduction and WC reduction were more frequent in the intervention group compared to the control group.

It may seem remarkable that high-risk subjects of the intervention group gained BW, BMI and WC, on average. First, the context on Reunion Island is obesogenic, with a high level of environmental exposure (in particular low socio-economic conditions), and prevalent unhealthy lifestyle behavior [34,35]. Second, the aging (+9 years) of the entire *fixed cohort* [16] may have contributed to the natural weight gain, even more in a population with prevalent total obesity at baseline (43%) and a large majority (74%) of women of childbearing age (18–40 years). Third, individuals were selected in a real-life setting (after screening at home) without applied eligibility criteria on their propensity to participate in the proposed workshops. Actually, almost half of the high-risk subjects of the intervention group participated in the workshops of the lifestyle program [18].

The long-term effects are encouraging, in view of the modest one-year mean change observed with the short-term effectiveness analysis performed in 2004 [18] showing a 0.26 effect-size (Cohen’s d) on BW.

In the literature on diabetes prevention by lifestyle intervention implemented in a community-based setting, very few studies report long-term post-intervention outcomes. In the GOAL study [36], 28 months after an 8-month intervention-period, mean changes from inclusion were: BW, -1.0 kg p = 0.003; BMI, -0.5 kg/m² p = 0.001; WC, +0.1 cm NS. In the SDPI-DP demonstration project [37], 24 months after a 12-month overall intervention period, mean change from inclusion in BW was -1.1 kg (significantly different from 0). These two intervention studies show better improvements than the intervention group of the REDIA-prev1 cohort study. However, the post-intervention length was three times shorter than ours (24–28 months versus 84 months, respectively). Moreover, the two populations had a higher baseline risk level (mean-BMI = 32.6–35.2 kg/m² versus median BMI = 29.3 kg/m², respectively) which increases probability of regression to the mean without a control group to deal with [38].

In our intervention group, the direction and magnitude of the mean change in WC are comparable to the Diabetes Prevention Program Outcomes Study (DPPOS) findings restricted to the subset of subjects with impaired glucose tolerance, aged 25–44 and exposed to the DPP lifestyle intervention [7]. In this DPPOS subgroup (N = 233), the mean change in WC assessed nine years after randomization, was ~+2.0 cm (graphical determination from web appendix) comparable with the value found in our study (+1.9 cm on available dataset, +2.1 cm on imputed dataset -Table 4).

### Study limitations

Our study has several limitations. The attrition of the cohort (42%) is high, but similar to the attrition of a cluster-controlled trial conducted in a district in Tehran for T2D primary prevention using community-based lifestyle intervention [39]. In the REDIA-prev1 cohort study, attrition was dealt with via multiple imputation of missing data under *MAR assumption* (i.e., ‘when the probability of missing data on a variable Y is related to some other measured variables in the analysis model but not to the values of Y itself’ [32]). Even if we cannot exclude the *missing not at random (MNAR) assumption* (i.e., ‘when the probability of missing data on a variable Y is related to the values of Y itself, even after controlling for other variables’ [32]), MAR seems plausible for two reasons. First, we included in the imputation models the subject’s participation in the trial completion examination (yes/no), which could be a short-term indicator of the propensity to be followed up on in the long-term (yes/no). The addition of this observed status (which is predictive of cohort attrition) as an auxiliary variable enhances the plausibility of the MAR assumption [32]. Second, in the intervention group, the selection biases towards female gender (exhibiting greater participation in follow-up than males) and age (lesser participation in the youngest) seem a consistent mechanism of attrition. These selection biases have already been observed in two previous Reunion Island population-based surveys with an enrolment step at home [17,40], whatever the health condition of interest (communicable / non-communicable diseases) and the context of research (epidemic / non-epidemic). As other baseline characteristics, gender and age were included in the imputation models to increase confidence in the MAR assumption. Furthermore, the mean baseline outcome level was not modified by attrition, including after stratification of the exposure group (Table 2).

Missing data are inevitable when following a sample of young adults selected from a population, within vulnerable districts, at home, over almost a decade. In French vulnerable districts, more than one third of inhabitants lived in another place five years before [41].

To decrease the impact of cohort attrition on available data, future intervention follow-up studies should involve participants in the research process, particularly in the context of vulnerable populations. The use of repeated contact and measurement could provide a direct individual benefit to health for the participants (by medical information delivery), and numerous intermediate data for evaluation (i.e., repeated measures design).

The statistical analysis followed an intention-to-treat principle, to test the hypothesis of the spread of protective healthy behaviour from intervention participants towards non-participants within intervention group since inclusion. In this intention-to-treat comparison, the use of a control group exposed to a minimal intervention (I- medical and nutritional information delivered after screening at inclusion; II- medical examination at trial completion) may decrease the contrast in the evaluation, and thus reduce the effect-size.

The three-month intergroup difference in follow-up length may have little consequence on the intention-to-treat analysis when studying changes during a long period of nine years (particularly in the process of latent metabolic chronic diseases).

The small sample size and the lack of both a gold standard for diabetes diagnosis (oral glucose tolerance test, fasting plasma glucose test) and annual testing for hazard ratio estimation inhibit the effective evaluation of diabetes incidence rate. This question has already been addressed by the SDPI-DP program [37]: the crude diabetes incidence among participants who achieved all sessions was significantly lower than that of other participants (~3.5% per year vs 7.5% per year, p<0.0001). However, the aim of our combined lifestyle intervention was the *primary* prevention of T2D by risk factor reduction [18]. First, the exclusion of prevalent diabetes cases using a stringent cut-off (HbA1c < 6.0% [2]) focused the study on prevention. Second, the use of adiposity outcomes (BW, BMI, WC) to evaluate effectiveness of lifestyle modifications, on a high-risk population mainly selected on the same anthropometric criteria (overweight / total obesity, central obesity), was consistent with a primary prevention goal. Third, the relationship between lifestyle behaviour improvement and glycaemic regulation had been shown to be mediated by BW change in high-risk population [6,42,43]. Fourth, the risk factor reduction is promising, assuming that modifiable factors relating to BW, diet and physical activity, are more likely to influence glycaemic status than genetic predispositions during a behavioural weight-reduction program [44].

### Study strengths

This cohort study is a *quasi-experiment with multiple groups*, ‘a mixed design combining elements of both internal and external comparisons, which enhances the potential for making a causal inference’ [16]. In particular, the control group allows adjustment for regression to the mean [38], an expected statistical phenomenon in populations with a high level of baseline outcome value.

We used a pragmatic strategy to deal with cohort attrition in statistical analyses. This strategy comprised four steps: *i-* exclude missing data at follow-up (Fig 1) for a naïve analysis on available dataset (Table 4) producing estimates under missing completely at random assumption; *ii-* support the MAR assumption by identifying any explanatory variables of missingness mechanism in data (Tables 1, 2 and 3) or other local studies using the same individuals selection process (experience of the research-team in the field); *iii-* include the explanatory variables and remaining baseline characteristics (including analysis model covariates) in the multiple imputation models to make the MAR assumption more plausible; *iiii-* impute missing data (Fig 1) for a non-biased analysis under MAR to confirm or not the findings of the first analysis on available dataset (Table 4).

The individual mean of the two successive WC measurements was used to calculate nine-year change. This process may have decreased the impact of measurement error and thus improved the quality of outcome assessment.

The outcomes assessment at nine years from baseline is original, since in the field of real-world T2D prevention by lifestyle intervention [10,36–37] the follow-up has not been investigated so far away.

### Conclusions: implications for research and public health

In the context of low socio-economic communities, our data support the assumption of long-term effect of lifestyle interventions targeting total obesity and central obesity two major drivers of T2D. Further studies are needed to investigate the spread of protective nutritional behaviour from participant to offspring and other persons in the local social network. We thus plan an extension of the original cohort to all household members in the next research step. Behavioural mechanisms that explain obesity diffusion in a social network [45] could be managed with a prevention goal.

## Supporting Information

S1 Checklist STROBE for cohort studies. (DOCX)

S1 Appendix Adherence to the lifestyle intervention. Three figures for three definitions.
(DOCX)

S2 Appendix Improvement in lifestyle after trial completion. Data are reported improvements in physical activity and diet occurred after the end of the lifestyle intervention trial. These results are from a subset of high-risk participants within the REDIA-prev1 cohort study.
(DOCX)

S1 Table Nine-year changes in body weight (BW), body mass index (BMI) and waist circumference (WC): non-adjusted estimates. (DOCX)

## Abbreviations

BMI: body mass index
BW: body weight
HbA1c: glycated haemoglobin A1c
MAR: missing at random
T2D: type 2 diabetes
WC: waist circumference
Δ: intergroup difference in mean change
