# Supplementary material for: Long-Term Effectiveness of a Lifestyle Intervention for the Primary Prevention of Type 2 Diabetes in a Low Socio-Economic Community – An Intervention Follow-Up Study on Reunion Island
Source: PLoS One. 2016 Jan 5;11(1):e0146095. doi: 10.1371/journal.pone.0146095 (PMC4701421; doi:10.1371/journal.pone.0146095)
Supplement: S1 Checklist — (DOCX) [file pone.0146095.s001.docx]

Long-Term Effectiveness of a Lifestyle Intervention for the Primary Prevention of Type 2 Diabetes in a Low Socio-Economic Community

– an Intervention Follow-up Study on Reunion Island

*(The REDIA-prev1 cohort study)*

**N/A**: not applicable

|  | Item No. | Recommendation | Fulfilled | Reported in section |
| --- | --- | --- | --- | --- |
| **Title and abstract** | 1 | (*a*) Indicate the study’s design with a commonly used term in the title or the abstract | ⌧ | - Title.  - Abstract. |
|  |  | (*b*) Provide in the abstract an informative and balanced summary of what was done and what was found | ⌧ | Abstract |
| Introduction | | | | |
| Background/rationale | 2 | Explain the scientific background and rationale for the investigation being reported | ⌧ | Introduction |
| Objectives | 3 | State specific objectives, including any prespecified hypotheses | ⌧ | (end of) Introduction |
| Methods | | | | |
| Study design | 4 | Present key elements of study design early in the paper | ⌧ | Study design and settings |
| Setting | 5 | Describe the setting, locations, and relevant dates, including periods of recruitment, exposure, follow-up, and data collection | ⌧ | Study design and settings |
| Participants | 6 | (*a*) Give the eligibility criteria, and the sources and methods of selection of participants. Describe methods of follow-up | ⌧ | - Population.  - Study design and settings.  - (beginning of) Data collection. |
|  |  | (*b*) For matched studies, give matching criteria and number of exposed and unexposed | **N/A** | - |

|  | Item No. | Recommendation | Fulfilled | Reported in section |
| --- | --- | --- | --- | --- |
| Variables | 7 | Clearly define all outcomes, exposures, predictors, potential confounders, and effect modifiers. Give diagnostic criteria, if applicable | ⌧ | - Outcomes.  - Intervention & S1 Appendix.  - Statistical analysis. |
| Data sources/ measurement | 8* | For each variable of interest, give sources of data and details of methods of assessment (measurement). Describe comparability of assessment methods if there is more than one group | ⌧ | Data collection |
| Bias | 9 | Describe any efforts to address potential sources of bias | ⌧ | - Usual diet assessment.  - Outcomes.  - Statistical analysis. |
| Study size | 10 | Explain how the study size was arrived at | ⌧ | Minimum detectable difference: prior calculation |
| Quantitative variables | 11 | Explain how quantitative variables were handled in the analyses. If applicable, describe which groupings were chosen and why | ⌧ | Statistical analysis |
| Statistical methods | 12 | (*a*) Describe all statistical methods, including those used to control for confounding | ⌧ | Statistical analysis |
|  |  | (*b*) Describe any methods used to examine subgroups and interactions | In next publication project | |
|  |  | (c) Explain how missing data were addressed | ⌧ | - Statistical analysis.  - Figure 1. |
|  |  | (*d*) If applicable, explain how loss to follow-up was addressed | ⌧ | Figure 1 |
|  |  | (*e*) Describe any sensitivity analyses | ⌧ | Statistical analysis |

|  | **Item No.** | **Recommendation** | **Fulfilled** | Reported in section |
| --- | --- | --- | --- | --- |
| Results |  | | | |
| Participants | 13* | (a) Report numbers of individuals at each stage of study—eg numbers potentially eligible, examined for eligibility, confirmed eligible, included in the study, completing follow-up, and analysed | ⌧ | - Figure 1.  - Selection of participants. |
|  |  | (b) Give reasons for non-participation at each stage | ⌧ | Figure 1 |
|  |  | (c) Consider use of a flow diagram | ⌧ | Figure 1 |
| Descriptive data | 14* | (a) Give characteristics of study participants (eg demographic, clinical, social) and information on exposures and potential confounders | ⌧ | - Selection of participants.  - Tables 1 & 2. |
|  |  | (b) Indicate number of participants with missing data for each variable of interest | ⌧ | Tables 1 & 2 ** |
|  |  | (c) Summarise follow-up time (eg, average and total amount) | ⌧ | Selection of participants |
| Outcome data | 15* | Report numbers of outcome events or summary measures over time | ⌧ | Table 4 (Binary outcomes on available dataset) |
| Main results | 16 | (*a*) Give unadjusted estimates and, if applicable, confounder-adjusted estimates and their precision (eg, 95% confidence interval). Make clear which confounders were adjusted for and why they were included | ⌧ | - S1 Table.  - Table 4.  - Nine-year changes in BW, BMI and WC: continuous outcomes. |
|  |  | (*b*) Report category boundaries when continuous variables were categorized | ⌧ | Table 4 |
|  |  | (c) If relevant, consider translating estimates of relative risk into absolute risk for a meaningful time period | **N/A** | - |
| Other analyses | 17 | Report other analyses done—eg analyses of subgroups and interactions, and sensitivity analyses | Table 3  S2 Appendix | |

|  | **Item No.** | **Recommendation** | **Fulfilled** | Reported in section |
| --- | --- | --- | --- | --- |
| Discussion | | | | |
| Key results | 18 | Summarise key results with reference to study objectives | ⌧ | (beginning of) Discussion |
| Limitations | 19 | Discuss limitations of the study, taking into account sources of potential bias or imprecision. Discuss both direction and magnitude of any potential bias | ⌧ | Study limitations |
| Interpretation | 20 | Give a cautious overall interpretation of results considering objectives, limitations, multiplicity of analyses, results from similar studies, and other relevant evidence | ⌧ | Discussion |
| Generalisability | 21 | Discuss the generalisability (external validity) of the study results | ⌧ | Conclusion |
| Other information | | | | |
| Funding | 22 | Give the source of funding and the role of the funders for the present study and, if applicable, for the original study on which the present article is based | ⌧ | Not in the manuscript.  Online submission. |

*Give information separately for exposed and unexposed groups.

** In an alternative presentation of results, we detailed the number of participants with available data for each variable of interest.
