# Supplementary material for: Long-Term Effectiveness of a Lifestyle Intervention for the Primary Prevention of Type 2 Diabetes in a Low Socio-Economic Community – An Intervention Follow-Up Study on Reunion Island
Source: PLoS One. 2016 Jan 5;11(1):e0146095. doi: 10.1371/journal.pone.0146095 (PMC4701421; doi:10.1371/journal.pone.0146095)
Supplement: S1 Appendix — Three figures for three definitions. (DOCX) [file pone.0146095.s002.docx]

**How many participants really adhered to the program?**

Several definitions of adherence can be used for a complex intervention like the REDIA-prev1 prevention program. Consequently, several estimations of adherence rate can be calculated.

1. **A broad definition:**

Before starting the workshops, the first step in the intervention was: *‘Screening at home for the type 2 diabetes risk factors’* followed by *‘Immediate delivery of medical information and guidelines to high-risk subjects’*. Both components were aimed at inducing the demand for prevention in a vulnerable population possibly unaware of and/or uninformed about diabetes risk factors. Since all the high-risk subjects were concerned with these two processes, a preliminary estimate of adherence within the intervention group would be **100%**.

1. **A conventional definition:**

More conventionally, a second estimate of adherence could be calculated using as numerator the number of high-risk screened and informed subjects who participated in the workshops (outdoor/indoor physical activities, healthy eating workshops, or support groups); this calculation gives an adherence rate of almost **50%** (Favier et al., 2005).

1. **An extended definition including empowerment:**

In a third definition of adherence, the figure of 50% could be updated by taking into account the workshop non-participants who were screened and informed and reported improvements in their lifestyles over the trial period. In this last case, the adherence rate increased to around **70%** (Favier et al., 2005).

**Reference:**

Favier F, Fianu A, Naty N, Le Moullec N, Papoz L. Essai de prévention primaire du diabète de type 2 et du syndrome métabolique à la Réunion [Trial for the primary prevention of type 2 diabetes mellitus and the metabolic syndrome in La Réunion]. Revue Médicale de l’Assurance Maladie. 2005;36:5-13. Available: https://epidemiologie-france.aviesan.fr/content/download/91002/3639089/version/1/file/

Favier_Revue_Med_Assurance_Mal_2005.pdf
