# Supplementary material for: Long-Term Effectiveness of a Lifestyle Intervention for the Primary Prevention of Type 2 Diabetes in a Low Socio-Economic Community – An Intervention Follow-Up Study on Reunion Island
Source: PLoS One. 2016 Jan 5;11(1):e0146095. doi: 10.1371/journal.pone.0146095 (PMC4701421; doi:10.1371/journal.pone.0146095)
Supplement: S2 Appendix — Data are reported improvements in physical activity and diet occurred after the end of the lifestyle intervention trial. These results are from a subset of high-risk participants within the REDIA-prev1 cohort study. (DOCX) [file pone.0146095.s003.docx]

**Did the participants change their eating habits, and did they really perform more exercise?**

**Data collection:** A home-based interview was conducted by a dietician (2010-2011), during which participants had the opportunity to report major changes in their lifestyle after trial completion (i.e., after July 2003). In this retrospective time-window of seven years on average, several major successive changes could have occurred. Each one of these major changes was documented by a start-date and an end-date (otherwise the change was still ongoing and therefore censored at the date of the dietician’s visit). For history of physical activity, only sport activity was investigated. There were no questions concerning intensity or frequency. Major increases in physical activity were self-rated. For diet history, questions focused on changes in portion size for the following food categories: sweets, rice, other starch, butter, oil, cheese, other milk products, fish/sea food, meat, delicatessen, fried foods, fruits and vegetables, beans, alcohol.

**Data management:** We classified each reported change in portion size (increase/decrease) as a major protective change against obesity (or not), based on the National Nutrition and Health Program guidelines and on local practices. For example, an increase in fruit portion size was classified as a protective change, whereas a decrease in fruit portion size was not classified as a protective change. The cumulative count of changes was calculated as follows: from zero to up to two for the number of major increases in physical activity, and from zero to up to fifteen for the number of major protective changes in food portion size. Lastly, the respective cumulative duration of changes (in months) was also calculated.

**Blinding:** Data management was conducted without knowledge of individual variation in adiposity and unbeknown to exposure group (intervention/control).

**Statistical analysis:** The comparison of distribution between the intervention group and the control group used a chi-square test for class variables, or a bivariate zero-inflated Poisson (zip) regression model for count variables and duration variables. The zip regression model allowed us to estimate the raw incidence rate ratio (IRR) and its 95% confidence interval (95%CI) for significant effect at p <0.05. Statistical analysis was performed using Stata version 10.

**Selection of participants:** Within the REDIA-prev1 cohort study (N_0_ = 445 at inclusion), the subset used for this analysis (N_2_ = 213) comprised subjects available for a second home-visit (dietician’s visit) conducted a few days to a few weeks after the first home-visit (nurse’s visit) at follow-up (N_1_ = 259). Compared to non-participants (N_2’_ = 232), participants in the second home-visit (N_2_ = 213) were: older (respectively, median age: 31.4 yrs versus 32.7 yrs, p<0.05), more often women (respectively, 66.8% versus 76.1%, p<0.04), with less upper value of baseline HbA1c = [5.5% to 5.9%] (respectively, 34.5% versus 22.5%, p=0.005).

**Main results:** In both groups, a majority of participants did not report important improvements in lifestyle after the end of the trial (S2 Table).

**S2 Table. Reported improvements in physical activity and diet occurred after the lifestyle intervention trial: Data are collected from a subset of high-risk participants within the REDIA-prev1 cohort study.**

| **Reports at follow-up for the 7-year period (2003 → 2010-2011)** | **Intervention**  **group** | | **Control**  **group** | | **p** |
| --- | --- | --- | --- | --- | --- |
| Subset size | 110 |  | 103 |  | - |
|  |  |  |  |  |  |
| ***Major increases in physical activity*** |  |  |  |  |  |
| **Cumulative count** (class) |  |  |  |  |  |
| None | 60 | (54.5) | 56 | (54.4) | 0.635 |
| One | 44 | (40.0) | 44 | (42.7) |  |
| Two | 6 | (5.5) | 3 | (2.9) |  |
| **Cumulative duration** (percentile) |  |  |  |  |  |
| P50 (months) | 0 |  | 0 |  | <0.001 |
| P75 (months) | 12 |  | 6 |  |  |
| P90 (months) | 36.5 |  | 24 |  |  |
| P95 (months) | 56 |  | 38 |  |  |
| Max (months) | 87 |  | 62 |  |  |
|  |  |  |  |  |  |
| ***Major protective changes in food portion size*** |  |  |  |  |  |
| **Cumulative count** (percentile) |  |  |  |  |  |
| P50 (months) | 0 |  | 1 |  | 0.315 |
| P75 (months) | 4 |  | 6 |  |  |
| P90 (months) | 8.5 |  | 9 |  |  |
| P95 (months) | 10 |  | 9 |  |  |
| Max (months) | 15 |  | 12 |  |  |
| **Cumulative duration** (percentile) |  |  |  |  |  |
| P50 (months) | 0 |  | 0 |  | <0.001 |
| P75 (months) | 10 |  | 12 |  |  |
| P90 (months) | 51.5 |  | 44 |  |  |
| P95 (months) | 82 |  | 70 |  |  |
| Max (months) | 94 |  | 87 |  |  |

Data are: number (column percentage); P50, median; P75, third quartile; P90, 90^th^ percentile; P95, 95^th^ percentile; Max, maximum; and pvalue of statistical tests comparing the distribution of variables between the two groups.

There was no difference between groups in the cumulative count of major increases in physical activity (p = 0.635) or that of protective changes in food portion size (p = 0.315). The cumulative duration was higher in the intervention group than in the control group (reference), for both major increases in physical activity (IRR = 1.52, 95%CI = 1.38 to 1.67, p<0.001) and major protective changes in food portion size (IRR = 1.20, 95%CI = 1.11 to 1.30, p<0.001). Control for gender, age and HbA1c at inclusion in multivariate models did not modify the interpretation of results.

**Discussion:** Findings show that participants in the intervention group initiated, after trial-completion, improvements in lifestyle (physical activity and diet) of longer duration than the control group. These retrospective data on reported lifestyle improvements were collected from a self-selected subset of participants representing 48% (213/445) of the overall sample. Inference to the entire REDIA-prev1 Cohort population should thus be made with caution.

**Bibliography:**

National guidelines:

Hercberg S. Programme National Nutrition Santé [National Nutrition and Health Program]. Arch Pediatr. 2003;10 Suppl 1:54s-56s.

Local practices:

Favier F, Rachou E, Ricquebourg M, Fianu A. Comportements alimentaires et activité physique des réunionnais (enquête RECONSAL) [Diet and physical activity behaviours of Reunion Island inhabitants (The RECONSAL Study)]. INSERM, ORS La Réunion. 2002. Available: http://www.ors-reunion.org/IMG/file/etudes/RECONSAL_2002.pdf

* * *
