# Supplementary material for: Long-Term Effectiveness of a Lifestyle Intervention for the Primary Prevention of Type 2 Diabetes in a Low Socio-Economic Community – An Intervention Follow-Up Study on Reunion Island
Source: PLoS One. 2016 Jan 5;11(1):e0146095. doi: 10.1371/journal.pone.0146095 (PMC4701421; doi:10.1371/journal.pone.0146095)
Supplement: S1 Table — (DOCX) [file pone.0146095.s004.docx]

**S1 Table. Nine-year changes in body weight (BW), body mass index (BMI) and waist circumference (WC): non-adjusted estimates**

| **Outcomes** | **Analysis on dataset** | **Intervention group** | | | | **Control group** | | | | **Intention-to-treat analysis: intervention versus control** | | | |
| --- | --- | --- | --- | --- | --- | --- | --- | --- | --- | --- | --- | --- | --- |
| *Continuous* |  | **N** | **Mean** | **95% CI** | **p** | **N** | **Mean** | **95% CI** | **p** | **N** | **Δ** | **95% CI** | **p** |
| **BW (kg)** | available | 125 | +3.0 | +1.6 to +4.4 | <0.001 | 134 | +5.1 | +3.8 to +6.5 | <0.001 | 259 | -2.2 | -4.1 to -0.2 | 0.030 |
| **BW (kg)** | imputed | - | +3.1 | +1.5 to +4.8 | <0.001 | - | +5.2 | +3.6 to +6.9 | <0.001 | - | -2.1 | -4.5 to +0.3 | 0.085 |
| **BMI (kg/m²)** | available | 125 | +1.14 | +0.59 to +1.69 | <0.001 | 132 | +1.98 | +1.45 to 2.51 | <0.001 | 257 | -0.84 | -1.61 to -0.08 | 0.031 |
| **BMI (kg/m²)** | imputed | - | +1.20 | +0.60 to +1.81 | <0.001 | - | +1.99 | +1.37 to 2.61 | <0.001 | - | -0.79 | -1.66 to +0.08 | 0.076 |
| **WC (cm)** | available | 124 | +1.1 | -0.4 to +2.6 | 0.161 | 134 | +4.3 | +2.8 to +5.8 | <0.001 | 258 | -3.2 | -5.3 to -1.1 | 0.003 |
| **WC (cm)** | imputed | - | +2.0 | +0.2 to +3.9 | 0.030 | - | +4.5 | +2.9 to +6.2 | <0.001 | - | -2.5 | -4.9 to -0.1 | 0.040 |
| *Binary* | **Dataset** | **n/N** | **Pr** | **95% CI** | **p** | **n/N** | **Pr** | **95% CI** | **p** | **N** | **RR** | **95% CI** | **p** |
| **BW loss** | available | 40/125 | 0.32 | 0.24 to 0.41 | <0.001 | 28/134 | 0.21 | 0.14 to 0.29 | <0.001 | 259 | 1.53 | 1.01 to 2.32 | 0.045 |
| **BW loss** | imputed | - | 0.35 | 0.27 to 0.43 | <0.001 | - | 0.25 | 0.17 to 0.32 | <0.001 | - | 1.44 | 0.98 to 2.12 | 0.062 |
| **BW loss ≥ 5%** | available | 21/125 | 0.17 | 0.11 to 0.25 | <0.001 | 12/134 | 0.09 | 0.05 to 0.15 | <0.001 | 259 | 1.88 | 0.96 to 3.65 | 0.064 |
| **BW loss ≥ 5%** | imputed | - | 0.19 | 0.13 to 0.26 | <0.001 | - | 0.12 | 0.06 to 0.18 | <0.001 | - | 1.61 | 0.89 to 2.91 | 0.116 |
| **BMI reduction** | available | 40/125 | 0.32 | 0.24 to 0.41 | <0.001 | 27/132 | 0.20 | 0.14 to 0.28 | <0.001 | 257 | 1.56 | 1.03 to 2.39 | 0.038 |
| **BMI reduction** | imputed | - | 0.35 | 0.27 to 0.43 | <0.001 | - | 0.24 | 0.17 to 0.32 | <0.001 | - | 1.45 | 0.99 to 2.12 | 0.060 |
| **WC reduction** | available | 55/124 | 0.44 | 0.35 to 0.54 | <0.001 | 36/134 | 0.27 | 0.20 to 0.35 | <0.001 | 258 | 1.65 | 1.17 to 2.32 | 0.004 |
| **WC reduction** | imputed | - | 0.44 | 0.36 to 0.52 | <0.001 | - | 0.30 | 0.22 to 0.37 | <0.001 | - | 1.47 | 1.07 to 2.03 | 0.017 |

Pr = proportion (same as Table 2). RR = relative risk (intervention group as exposure category versus control group as reference category). BW loss ≥ 5% of the inclusion measurement. 95% CI = 95% confidence interval. Continuous outcome within group = follow-up measurement minus inclusion measurement. Δ = intergroup difference in mean change (intervention minus control). p for test with (H0: mean = 0) or (H0: Δ = 0) or (H0: Pr = 0) or (H0: RR = 1) according to outcome and comparison. Missing data were imputed under MAR assumption with MICE method implemented in Stata 10 (ice and Micombine packages). Variables included in the imputation models: baseline characteristics, exposure-group, participation in the medical visit at trial completion (yes/no), and the shifted log transformation of BW and WC at follow-up, respectively. Forty imputed datasets were generated from an original dataset of 432 observations.
